# Cognitive Impairment in Chinese Patients with Sporadic Amyotrophic Lateral Sclerosis

**DOI:** 10.1371/journal.pone.0137921

**Published:** 2015-09-14

**Authors:** Bo Cui, Liying Cui, Jing Gao, Mingsheng Liu, Xiaoguang Li, Caiyan Liu, Junfang Ma, Jia Fang

**Affiliations:** 1 Department of Neurology, Peking Union Medical College Hospital, Chinese Academy of Medical Sciences, Beijing, China; 2 Neurosciences Center, Chinese Academy of Medical Sciences, Beijing, China; Institute of Health Science, CHINA

## Abstract

**Background:**

It has reached a consensus that patients with amyotrophic lateral sclerosis (ALS) could display cognitive impairment characterized by executive dysfunction or even dementia, but cognitive spectrum of Chinese patients with ALS still waits to be documented.

**Methods:**

A total of 106 incident patients with sporadic ALS were enrolled and comprehensive neuropsychological tests covering memory, executive function, attention, language, and visuospatial function were administered to them. Neuropsychological performances of 76 age- and education- matched healthy controls were used for the purpose of classification and comparison.

**Results:**

106 patients were categorized into 4 subtypes:84 (79.2%) ALS with normal cognition (ALS-NC), 12 (11.3%) ALS with executive cognitive impairment (ALS-ECI), 5 (4.7%) ALS with non-executive cognitive impairment (ALS-NECI), and 5 (4.7%) ALS with frontotemporal lobe degeneration (ALS-FTLD). Under the same criteria, 2 (2.6%) and 1 (1.3%) healthy controls were diagnosed as ECI and NECI, respectively. The proportion of ECI was significantly higher in non-demented ALS than that in healthy controls, but it was not for NECI. Patients with ALS-FTLD had significantly severer bulbar function and older age than those with ALS-NC.

**Conclusion:**

Comorbid FTLD occurred in around 5% of Chinese sporadic ALS cases. Different genetic background and unique age distribution of Chinese ALS patients might be the reasons for the relatively low rate of comorbid FTLD. Cognitive dysfunction, predominant but not exclusive in executive area, was present in around 16% of non-demented ALS patients.

## Introduction

Amyotrophic lateral sclerosis (ALS) is a progressive neurological disorder pathologically characterized by loss of motor neurons in spinal cord, brain stem and motor cortex of brain, leading to clinically variable combinations of dysarthria, muscle atrophy, limb weakness and pyramidal signs. Although predominantly manifested by motor symptoms, ALS belongs to a neurodegenerative spectrum involving multiple systems. Increasing researches found non-pyramidal features in ALS, including cognitive impairment, autonomic disorder and extrapyramidal signs [1, 2]. Specifically, patients with ALS could display similar pattern of executive dysfunction to that of frontotemproal lobe degeneration (FTLD), and the degree of it could vary from mild cognitive impairment to frank dementia [3]. Subsequently, evidence in favor of the relationship between ALS and FTLD has been accumulated, ranging from genetic basis to neuroimaging correlates [4, 5].

The diagnostic axis for ALS based on cognitivestatushas been established [6], and several studies have described cognitive spectrum of ALS patients in their own country [7–10]. Despite these attempts and advances in this field, problems remain to be solved: most relevant studies were carried out in Europe and North America, whereas the data of Chinese population turned to be rather limited. Actually, unique clinical characteristics and culture backgrounds of Chinese patients make it an reasonable assumption that they might also differ from Caucasian patients in cognitive profile[11, 12]. Possibly associated with onset type, function disability and progression rate, cognitive status in ALS seemed to be more than an academic interest [7–10], but the results concerning these relationships appeared to be inconclusive. Hence we perform a cross-sectional investigation as an effort to resolve these issues.

## Methods

All newly diagnosed cases of ALS and other types of motor neuron disease (MND) were consecutively enrolled in our ongoing registry platform for ALS and MND[11, 12]. Demographic and clinical information including age, gender, level of education, site of symptom onset, disease duration (defined as time lapse between symptom onset and time of diagnosis) were collected. Disease severity was assessed by the revised ALS functional rating scale (ALSFRS-R) [13]. To further analyze bulbar function, we applied swallowing subscale of the Amyotrophic Lateral Sclerosis Severity Scale (ALSSS) [14].

Patients included in this study should fulfill the revised El Escorial diagnostic criteria for clinically definite, probable and lab-supported probable ALS [15], while the data of those with family history of FTLD and ALS, history of other neurological conditions which could have an impact on neuropsychological assessments (major stroke, traumatic brain injury, learning disability and severe active epilepsy), alcohol-dependence, drug-dependence, severe depression, anxiety or other active mental illness, use of high-dose psychoactive medication, other mother language instead of Chinese Mandarin, and illiteracy were excluded from analysis. A total of 76 age and education-matched healthy controls (HC) were recruited and underwent the same neuropsychological batteries. They were all community residents and selected in accordance with the same exclusion criteria as ALS patients. This study was approved by the Research Ethics Committee of Peking Union Medical College Hospital. Patients visited our clinic accompanied by their legal guardians, and all the patients and HC were included after informed written consent had been obtained from themselves or their guardians, as set forth by the Declaration of Helsinki.

### Cognitive evaluation

Participants underwent a series of neuropsychological batteries. Selected tests were as follows: category and phonemic verbal fluency [16–18]; the Stroop ColorWord Task(SCWT) [19]; the Clock Drawing Test (CDT) [20]; paired associate word learning of the Clinical Memory Test (CMT)[21]; episodic memory of the modified Wechsler Memory Scale (WMS) [22]; the Symbol Digit Modalities Test (SDMT) [23]; digit span of the Wechsler Adult Intelligence Scale (WAIS) [24]; repetition and copy subsets ofthe Aphasia Battery of Chinese (ABC) [25]. Stroop interference effect (SIE) was assessed bya formula: SIE = Stroop C time/Stroop C correct number-Stroop B time/Stroop B correct number. For certain cases, complete set of the ABC was administered as a supplementary instruments for language variant dementia.

Depression and anxiety of patients were assessed by the Hamilton Depression Rating Scale (HDRS) [26] and Hamilton Anxiety Rating Scale (HARS)[27]. A score of >35 in HDRS and a score of ≥29 in HARS reached the exclusion criteria of severe depression and anxiety, respectively.

The batteries were administered within a week after the diagnosis was made, and it required about 2 hours to complete them. So, subjects could choose to take a short break or finish it at the same time next day if necessary, but the tests must be completed in a given sequence.

### Diagnostic criteria

We adopted the Neary criteria for frontotemporal dementia to diagnose FTLD [28], and the diagnosis of non-FTLD dementia was based on the Diagnostic and Statistical Manual of Mental Disorders-IV [29]. We then adopted a domain based classification for the rest patients without evidence of FTLD or dementia.2 standard deviations (SD) below the mean of HC was set as the cutoff value for each neuropsychological test except for the CDT (4 score method was adopted and ≤3 was set as the cut-off). At least 2 tests in one domain scored below the cutoffreached the threshold of impairment (cognitive domains and corresponding tests are shown in Table 1). Thus as long as non-demented subjects displayed impairment in executive function, they would be identified as ALS with executive cognitive impairment (ALS-ECI). And those with impairment in any non-executive domain but without executive dysfunction would be defined as ALS with non-executive cognitive impairment (ALS-NECI). Both ALS-ECI and ALS-NECI were regarded as ALS with cognitive impairment (ALS-CI). At last, the remaining patients were regarded as ALS with normal cognition (ALS-NC).

**Table 1:** Cognitive domains and corresponding neuropsychological tasks.

Cognitive domains	Neuropsychological tasks
Executive function	Phonemic verbal fluency
	Category verbal fluency
	Backward digit span of the Wechsler Adult Intelligence Scale
	the Stroop ColorWord Task
	the Clock Drawing Test
Attention	Forward digit span of the Wechsler Adult Intelligence Scale
	the Symbol Digit Modalities Test
Memory	Paired associate word learning of the Clinical Memory Test
	Episodic memory of the modified Wechsler Memory Scale
Language	Repetition subset of the Aphasia Battery of Chinese
	Category verbal fluency
Visuospatial function	Copy subset of the Aphasia Battery of Chinese
	the Clock Drawing Test

Case ascertainment, clinical diagnosis and categorization were supervised by 3 senior ALS specialists (L Cui, M Liu and X Li) and a senior dementia specialists (J Gao).

### Statistical Methods

We used means/median for continuous variables and proportion for category variables. In comparisons, one-way ANOVA and chi-square test were adopted for continuous and categorical variables, respectively. When data failed to meet criteria for parametric analysis, nonparametric analysis was applied instead. Bonferroni correction was applied to adjust α value in case of post hoc analysis and where multiple comparisons were undertaken. All tests were two tailed. Statistical significance was set at p<0.05. Statistical analyses were carried out using SPSS 11.5 (SPSS Inc).

## Results

### Selection of participants

In the period between September 1, 2013 and November 31, 2014, 150 incident patients were registered in our platform, and 124 patients fulfilled the revised El Escorial criteria for clinically definite, probable and lab-supported probable ALS. Then8of them were excluded (3 for positive family history, 1 for history of major stroke, 1 for history of cancer, 1 for mother language other than Chinese Mandarin and 2 for illiteracy). Among the remaining 116 patients,10 were not captured: 2 refused to participate in this study, while severe physical disability hindered the other 8 to complete enough batteries and consequently their cognitive diagnosis could not be made. Finally, 106 cases were included into analysis.

### Baseline demographic and clinical characteristics of patients

The mean age of captured patients at the time of diagnosis was 51.3±10.4 years (27.0–75.0 years), and 67 cases (63.2%) were men. 22 patients (20.8%) had bulbar-onset ALS, and the remaining 84 patients (79.2%) had spinal onset disease. Median time from symptom onset to diagnosis was 11 months (range 2–93). At baseline, median ALSFRS-R score was 42 (range 27–48), and median swallowing score of ALSSS was 10 (range 7–10). At the time of diagnosis, no one had gastrostomy or non-invasive ventilation. Compared to captured patients, the proportion of bulbar onset cases was significantly higher in non-captured patients. Baseline progression rate, ALSFRS-R and swallowing subscale of ALSSS were also significantly different between captured and non-captured cases (Table 2).

**Table 2:** Demographic and clinical characteristics of captured and non-captured ALS cases.

	Captured cases (n = 106)	Non-captured cases(n = 10)	p Value
Age at evaluation (years)	51.3±10.4	56.0±11.7	0.173
Educational level (years)	10.5±3.7	11.1±4.1	0.696
Gender (male, %)	63.2	50.0	0.462
Onset type (bulbar, %)	20.8	50.0	**0.036**
Disease duration (months)	14.2±11.6	14.9±12.2	0.726
Swallowing subscale of ALSSS	9.2±1.2	7.4±2.0	**0.002**
ALSFRS-R	40.8±4.6	31.4±8.1	**<0.001**
Progression rate	0.7±0.6	1.5±1.0	**0.011**

ALS, amyotrophic lateral sclerosis; ALSFRS-R, revised ALS functional rating scale; ALSSS, ALS severity scale; the disease progression rate was calculated according to the formula of (48-ALSFRS-R score)/disease duration(month).

Data were means±SD.

### Cognitive status and diagnosis of ALS and HC

First of all, 4 cases presented with prominent behavioral changes fulfilled the diagnostic criteria of behavioral variant FTLD (bvFTD), andone demented case was diagnosed as Semantic Dementia.

Then compared to HC, non-demented ALS performed significantly worse in category verbal fluency, backward digital span of the WAIS, episodic memory of modified WMS and the SDMT (Table 3). Also in these tests and the CDT, the proportion of participants scoring below cutoff was significantly higher in non-demented ALS than that in HC (Table 4).

**Table 3:** Comparison of demographic variables and neuropsychological performances between non-demented ALS and HC.

	non-demented ALS	n	HC	n	p Value
Age at evaluation (years)	50.7±10.1	101	50.3±10.2	76	0.815
Educational level (years)	10.6±3.6	101	10.7±3.2	76	0.945
Gender (male, %)	63.2	101	55.3	76	0.281
Phonemic verbal fluency	5.2±2.1	101	6.0±2.6	75	0.127
Category verbal fluency	15.8±3.7	101	18.0±4.1	76	**0.001**
Backward digital span of the WAIS	4.9±1.4	101	5.2±1.3	76	**0.033**
Stroop interference effect	0.7±0.4	87	0.6±0.3	75	0.107
Episodic memory of modified WMS	5.6±1.6	101	6.3±1.4	76	**0.003**
Paired associate word learning of the CMT	9.9±4.1	101	10.9±4.2	76	0.135
Forward digital span of the WAIS	7.8±1.1	101	7.9±1.1	76	0.741
the Symbol Digit Modalities Test	40.0±12.0	95	45.9±10.4	76	**0.003**
Errors in repetition of the ABC	1.6±1.6	101	1.6±1.8	72	0.976
Copy of the ABC	9.3±1.5	96	9.3±1.2	76	0.419

ABC, Aphasia Battery Chinese; ALS, amyotrophic lateral sclerosis; CMT, Clinical Memory Test; HC, healthy controls;WAIS, Wechsler Adult Intelligence Scale; WMS, Wechsler Memory Scale;stroop interference effect was calculated according to the formula of (Stroop C time/Stroop correct number-Stroop B time/Stroop correct number).

Data were means±SD.

**Table 4:** Comparison of proportion of impaired participants in each neuropsychological battery, cognitive domain and cognitive diagnosis between non-demented ALS and HC.

	non-demented ALS	HC	p Value
**Neuropsychological batteries**			
Phonemic verbal fluency	4/101	1/75	0.300
Category verbal fluency	5/101	0/76	**0.049**
Backward digital span of the WAIS	13/101	3/76	**0.040**
the Stroop Color Word Task	11/87	4/75	0.109
the Clock Drawing Test	26/101	9/74	**0.027**
Episodic memory of modified WMS	15/101	4/76	**0.041**
Paired associate word learning of the CMT	2/101	0/76	0.217
Forward digital span of the WAIS	10/101	4/76	0.258
the Symbol Digit Modalities Test	16/95	2/76	**0.003**
Repetition of the ABC	12/101	5/72	0.282
Copy of the ABC	12/96	12/76	0.536
**Cognitive domain**			
Executive function	12/101	2/76	**0.024**
Attention	3/101	0/76	0.130
Memory	0/101	0/76	N/A
Language	2/101	0/76	0.217
Visuospatial function	5/101	2/76	0.433
**Cognitive diagnosis**			
Executive cognitive impairment	12/101	2/76	**0.024**
Non-executive cognitive impairment	5/101	1/76	0.186
Cognitive impairment	17/101	3/76	**0.007**

ABC, Aphasia Battery Chinese; ALS, amyotrophic lateral sclerosis; CMT, Clinical Memory Test; HC, healthy controls;WAIS, Wechsler Adult Intelligence Scale; WMS, Wechsler Memory Scale.

N/A, chi-square could not be performed when numerators of both groups are zero.

Data were shown in the form of number of participants with impairment/total number.

**Fig 1:**
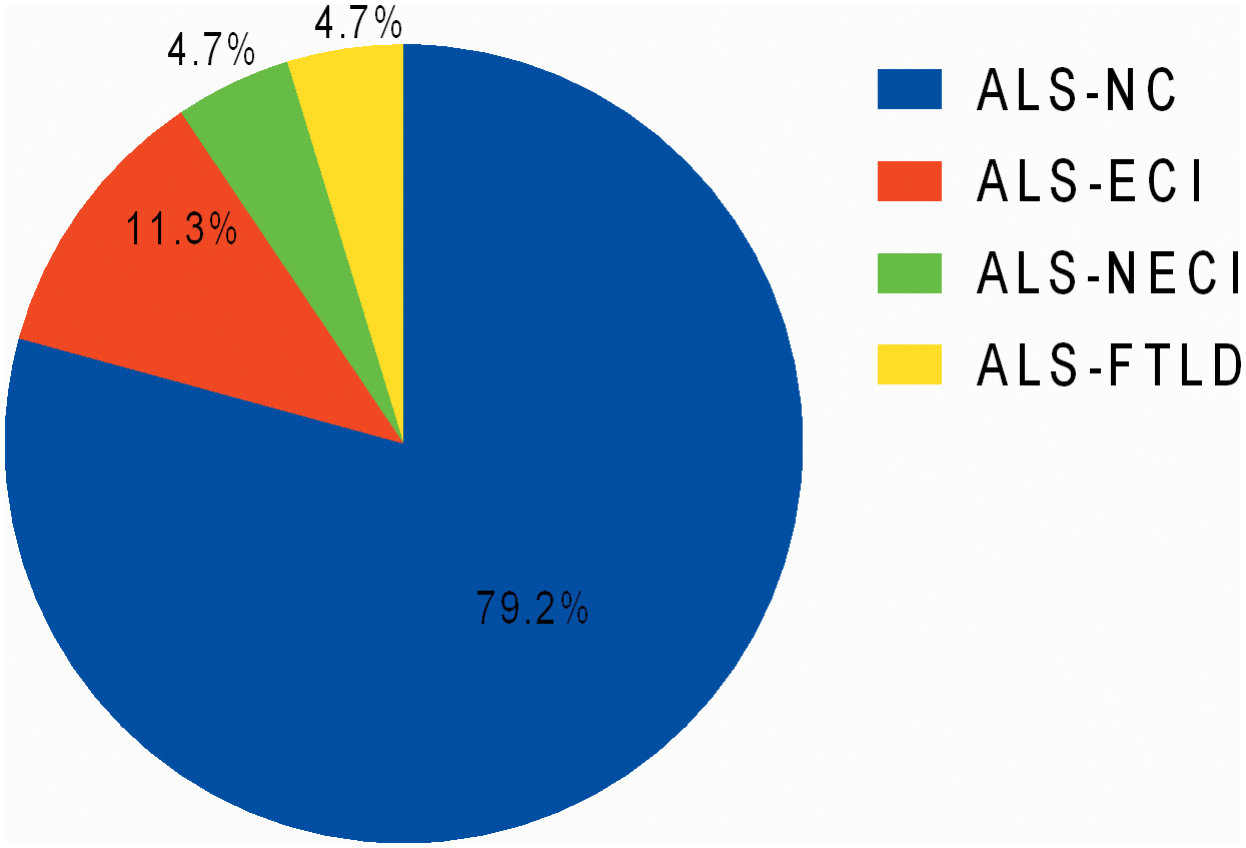
Categorization of Chinese patients with sporadic ALS according to cognitive status (n = 106). ALS, amyotrophic lateral sclerosis; ALS-ECI, ALS with executive cognitive impairment; ALS-FTLD, ALS with frontotemporal lobe degeneration; ALS-NC, ALS with normal cognition; ALS-NECI, ALS with non-executive cognitive impairment.

**Fig 2:**
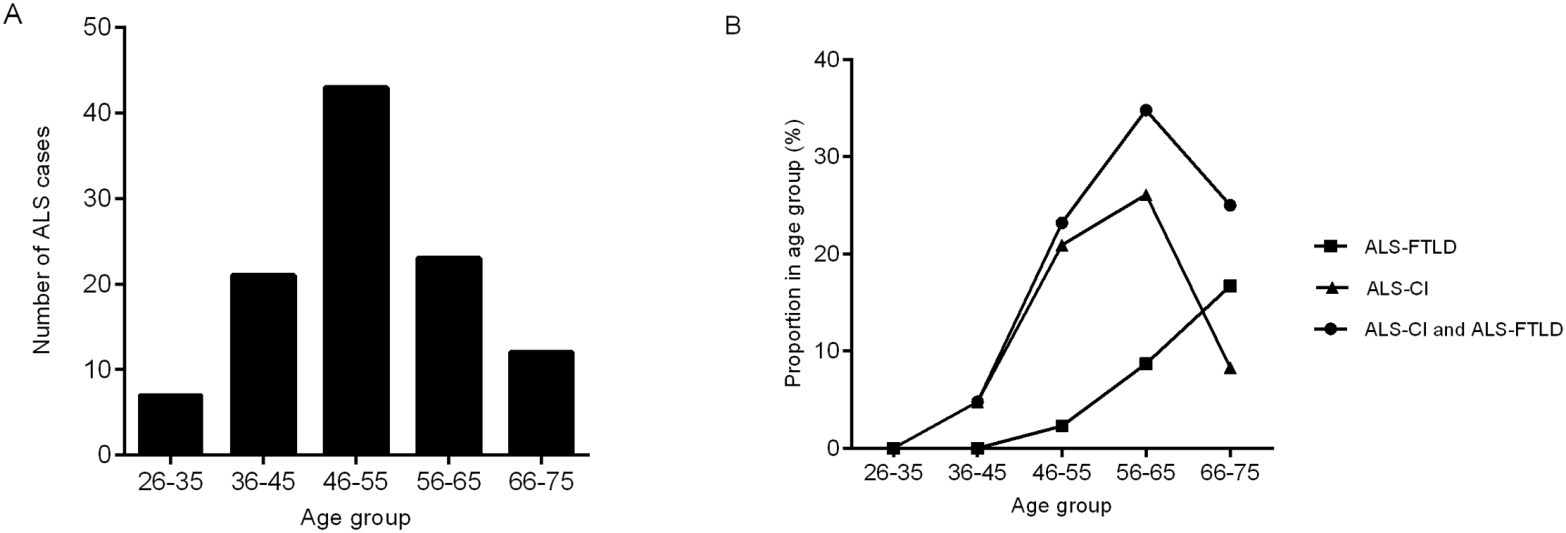
Age distribution of included ALS patients and the proportion of cognitive impairment and FTLD in each age group. (A) Age distribution of included ALS patients. (B) Prevalence of cognitive impairment in each age group. ALS, amyotrophic lateral sclerosis; ALS-CI, ALS with cognitive impairment; ALS-FTLD, ALS with frontotemporal lobe degeneration.

Hence under the criteria described above, 106 patients were categorized into 4 subtypes: 5 (4.7%) cases were diagnosed as ALS-FTLD, 12 (11.3%) asALS-ECI, 5(4.7%) as ALS-NECI, and remaining 84 (79.2%) as ALS-NC (Fig 1). Under the same criteria, 2 (2.6%) HC were diagnosed as ECI and one participant (1.3%) was diagnosed as NECI, thus the remaining 73 (96.1%) were cognitively normal. The proportion of participants with impairment in executive domain was significantly higher in non-demented ALS than that in HC (Table 4). Age distribution and proportion of patients with cognitive impairment in each age group were shown in Fig 2.

### Comparison of clinical and demographic variables among ALS-NC, ALS-ECI and ALS-FTLD

In the comparison of clinical and demographic variables, swallowing subscale of ALSSS and age at evaluation were significantly different among ALS-NC, ALS-ECI and ALS-FTLD. Post hoc analysis revealed that the differences between ALS-FTLD and ALS-NC survived Bonferroni correction (p = 0.001 and 0.015 respectively, adjusted α = 0.05/3 = 0.017) (Table 5).

**Table 5:** Demographic and clinical characteristics of ALS-NC, ALS-ECI and ALS-FTLD.

	ALS-NC (n = 84)	ALS-ECI (n = 12)	ALS-FTLD (n = 5)	p Value
Age at evaluation (years)	49.9±10.5	55.7±6.1	62.4±9.4	**0.007**
Educational level (years)	11.0±3.6	8.4±3.0	9.8±5.2	0.110
Gender (male, %)	64.2	58.3	60.0	0.911
Onset type (bulbar, %)	19.0	16.7	60.0	0.084
Disease duration (months)	14.0±12.4	12.2±6.5	20.0±7.9	0.201
Swallowing subscale of ALSSS	9.4±1.1	8.8±1.4	7.6±0.9	**0.002**
ALSFRS-R	41.1±4.1	39.5±5.4	39.4±7.4	0.713
Progression rate	0.7±0.6	0.8±0.6	0.4±0.3	0.337

ALS, amyotrophic lateral sclerosis; ALSFRS-R, revised ALS functional rating scale; ALSSS, ALS severity scale;ALS-NC, ALS with normal cognition; ALS-ECI, ALS with executive cognitive impairment; ALS-FTLD, ALS with frontotemporal lobe degeneration; the disease progression rate was calculated according to the formula of (48-ALSFRS-R score)/disease duration(months).

Data were means±SD.

## Discussion

Through neuropsychological approaches, cognitive deficit of different degrees was described in up to 50% of ALS patients in Europe and the US [8–10], and executive dysfunction occurred as the major type of it. Several studies assessed cognitive status of Chinese ALS patients and cognitive dysfunction was also noticed [30–32]. However, evaluation only by screening instruments would not be sufficient to make cognitive diagnosis case by case. Hence, we employed a series of neuropsychological batteries comprehensively covering several cognitive domains with emphasis on executive function. The demographic variables of this cohort were similar to those previously published by us and others [11,12,30–32]. Besides, its relatively large sample and high response rate added its credibility of truly reflecting characteristics of Chinese patients, although it is not a population-based design.

Under a relatively stringent cut-off suggested by previous literature (2SD below HC) [8, 10],cognitive impairment in ALS in this study appeared to be not as high as expected. Despite this, proportion of ECI remained to be significantly higher in non-demented ALS than that in HC, which was in favor of the existence of ALS-associated executive dysfunction. Unlike ECI, NECI turned not to be an ALS specific impairment in an Irish cohort [8]. In agreement with this conclusion, our results indicated the distribution of NECI was not significantly different between non-demented ALS and HC.

It is reasonable that the frequency of cognitive impairment varied among different investigations due to diverging diagnostic instruments and criteria. However, it is not for FTLD, diagnosis of which does not fully base on cognitive batteries. So we barely regarded it as a coincidence that the rates of comorbid FTLD fell below 5% in both our cohort and the Korean one[7], whereas the prevalence of it reached above 10% and up to 15% in Europe and the US[8–10]. Differences in ethnic and genetic backgrounds might be the clue to explain this disparity: responsible for 23.5%~47% familial ALS or FTLD and 4.1%~21.0% sporadic ALS in Europe and the US, C9ORF72 served as the bonding between ALS and FTLD with the most supporting evidence[33], but it appeared to be relatively rare in Eastern Asian population[7,34, 35]. Taking out familial cases and C9ORF72 cases from the Italian cohort, for instance, could slightly lower the rates of FTLD from 12.6% to 9.3% and 9.8%, respectively[10], which would be still higher than our sporadic cohort and the Korean one of no known mutation.

Age distribution could be another possible explanation for the relatively low prevalence of comorbid dementia in our cohort, considering it’s been proved that ALS-FTLD had an older onset age than those of normal cognition [10]. In the present study, rates of ALS-FTLD started to rise after 46 years old, whereas the rate of cognitive impairment was rather low in patients under 45 years old who accounted for 26% of the whole cohort. To the contrary, the mean age of Caucasians studies ranged from 58.8±14.4 to 67.0±9.9 [8–10], older than that of ours and the Korean one which was51.3±10.4 and 55.7±10.7, respectively. And this gap could hardly be attributed to patient selection, because it was in line with the difference of peak onset age proved before [6]. Based on these facts, it is likely that aging and neurodegeneration synergistically eroded cognitive reserve of ALS patients, or function of frontal and temporal lobes was inherently spared in early onset ALS.

The relationship between onset type and cognitive performance remained to be in dispute. Our results supported the connection between bulbar involvement and cognitive function: ALS-FTLD had significantly worse bulbar function evaluated by swallowing subscale of ALSSS than ALS-NC did, although the distribution of bulbar onset type was not significantly different between ALS-NC, ALS-ECI and ALS-FTLD. Moreover, there were more patients with bulbar onset and involvement in non-captured group than that in captured group, where lied a possibility that the rate of patients with CI or even comorbid FTLD was underestimated to some extent.

In the aspect of neuropsychology, category verbal fluency, backward digital span of the WAISand the CDT were proved to be sensitive for executive dysfunction among Chinese patients. It is because these batteries demanded participation of multiple frontostriatal circuits and thus any lesion among them would increase the difficulty of completing them [8]. Unlike previous studies[7–10], phonemic verbal fluency failed to be discriminating in our cohort, probably due to its inapplicability in population of education level below high school[17]. Besides, the SDMT demonstrated effectiveness in detecting deficit for ALS, and yet requiring perceptual processing speed, visual scanning, and memory, it is should not be viewed as a battery exclusively reflecting attention. Memory impairment is another unsettled problem with controversial results [7–10]. Paired associated word learning of the CMT was a sensitive task for amnesic cognitive impairment, but it seemed to be limited in detecting memory deficit in ALS. However, ALS scored significantly lower in episodic memory of modified WMS. This disparity might indicate that frontal lobe dysfunction rather than temporal lobe should be blamed for memory dysfunction of patients with ALS.

This study should be viewed with consideration of several limitations. Firstly, the conclusions drawn from a single center-based observation could be influenced by referral bias, which wait to be tested by future multicenter-based and population-based studies. Moreover, the system of neuropsychological evaluation still need to be improved and perfected. Verbal fluency test, for example, was not adjusted for patients with bulbar dysfunction, whose scores might be lowered consequently. And to avoid patients’ fatigue, we did not include the assessment of premorbid IQ, an important predictor of cognitive reserve, despite its minor impact on executive function[8]. Last but not least, data concerning respiratory involvement, such as forced vital capacity, was not acquired to analysis, and thus its contributions to cognitive findings could not be determined.

In summary, we presented the distribution of cognitive impairment in a Chinese ALS cohort, which was rather distinct from that of the US and Europe, especially in the frequency of FTLD. This phenomenon might be relevant to genetic and demographic differences. Future population-based researches might provide more accurate prevalence of cognitive syndrome in Chinese ALS patients, and confirmation to our results as well. Besides, physical disability and specific culture background should be taken into consideration in the design of neuropsychological evaluation, and we hope our research could supply information and experience for future ones.
